# Detection of Acute Radiation Sickness: A Feasibility Study in Non-Human Primates Circulating miRNAs for Triage in Radiological Events

**DOI:** 10.1371/journal.pone.0167333

**Published:** 2016-12-01

**Authors:** Naresh Menon, Claude J. Rogers, Agnes I. Lukaszewicz, James Axtelle, Marshleen Yadav, Feifei Song, Arnab Chakravarti, Naduparambil K. Jacob

**Affiliations:** 1 ChromoLogic LLC, Monrovia, California, United States of America; 2 Department of Radiation Oncology, The Ohio State University Comprehensive Cancer Center Columbus, Ohio, United States of America; ENEA Centro Ricerche Casaccia, ITALY

## Abstract

Development of biomarkers capable of estimating absorbed dose is critical for effective triage of affected individuals after radiological events. Levels of cell-free circulating miRNAs in plasma were compared for dose-response analysis in non-human primates (NHP) exposed to lethal (6.5 Gy) and sub-lethal (1 and 3 Gy) doses over a 7 day period. The doses and test time points were selected to mimic triage needs in the event of a mass casualty radiological event. Changes in miRNA abundance in irradiated animals were compared to a non-irradiated cohort and a cohort experiencing acute inflammation response from exposure to lipopolysaccharide (LPS). An amplification-free, hybridization-based direct digital counting method was used for evaluation of changes in microRNAs in plasma from all animals. Consistent with previous murine studies, circulating levels of miR-150-5p exhibited a dose- and time-dependent decrease in plasma. Furthermore, plasma miR-150-5p levels were found to correlate well with lymphocyte and neutrophil depletion kinetics. Additionally, plasma levels of several other evolutionarily and functionally conserved miRNAs were found altered as a function of dose and time. Interestingly, miR-574-5p exhibited a distinct, dose-dependent increase 24 h post irradiation in NHPs with lethal versus sub-lethal exposure before returning to the baseline level by day 3. This particular miRNA response was not detected in previous murine studies but was observed in animals exposed to LPS, indicating distinct molecular and inflammatory responses. Furthermore, an increase in low-abundant miR-126, miR-144, and miR-21 as well as high-abundant miR-1-3p and miR-206 was observed in irradiated animals on day 3 and/or day 7. The data from this study could be used to develop a multi-marker panel with known tissue-specific origin that could be used for developing rapid assays for dose assessment and evaluation of radiation injury on multiple organs. Furthermore this approach may be utilized to screen for tissue toxicity in patients who receive myeloablative and therapeutic radiation.

## Introduction

The lack of reliable biomarkers for evaluation of the extent of damage by radiation injury presents an impediment to medical decision-making regarding the triage and treatment of persons who might be at risk for developing Acute Radiation Sickness (ARS) following a nuclear/radiological accident or attack [1]. Radiation affects the hematologic, pulmonary, gastrointestinal, and other systems in various ways depending on the amount received. As such, ARS in humans follows a deterministic path whereby dose effects have distinct clinical outcomes: generally less than 2 Gy exhibit mild symptoms and hematological effects are prominent at doses between 2 and 6 Gy and progress slowly. Gastrointestinal effects are prominent at doses greater than 5–6 Gy and progress more rapidly, while neurovascular collapse ensues at doses greater than 10–20 Gy which occurs immediately [2,3]. Rapid and early diagnosis is essential as demonstrated by the dramatic increase in survival rates if treatments are administered within few days after exposure [4,5].

Hematological system being the most sensitive, lymphocytic depletion kinetics and evaluation of neutrophil counts, together with clinical symptoms, are commonly used to determine radiation exposure levels [6–8]. However, dose estimation based on hematological parameters requires multiple reading over days [9]. Also, variability of lymphocyte count among individuals decreases the accuracy of the diagnosis especially in individuals with compromised immune systems [10]. Further, dose estimation based on hematological parameters hold value only when exposure happens to the whole body or with significant coverage to the bone marrow. The gold standard dicentric chromosome (DC) assay in lymphocytes is highly technical and labor intensive, and is unlikely to be a viable for early triage [11]. Metabolites and several protein markers, such as C-reactive protein, amylase, and cytokines, have been investigated for their potential as biodosimeters [2–5,12,13]. However, inability to determine organ specific damage and fluctuations due to common differences in genetic and immune responses affect the readout, especially during partial body exposure to ionizing radiation. Therefore, there is an urgent need for identifying robust biomarkers that allow estimation of the absorbed dose and provide readout of physiological response of various organs.

miRNAs are small RNA molecules of 18–24 nt length, originally identified as regulators of gene expression and have been shown to regulate various physiological and pathological processes [14]. Studies over the last several years have demonstrated the potential of cellular as well as cell-free circulating miRNAs as biomarkers with diagnostic and predictive value for several human diseases and physiological states. There are over 2,000 miRNAs in mammalian cells and each cell type has a distinct signature with regard to their expression. For example, evolutionarily conserved sequences such as miR-451 and miR-150 are highly expressed in bone marrow [15], regulating hematopoiesis, and miR-126 and let-7 family members show high expression in lung [16,17]. miRNAs are released, via extracellular vesicles such as exosomes into the circulatory system, allowing minimally/non-invasive assessment of organ health and responses to various stimuli. These ‘circulating miRNA’ have the potential to provide diagnostic/prognostic information about an individual’s health, assayable from a simple blood draw or even a finger prick. Furthermore, being small, abundant, and protected from degradation in extracellular vesicles, circulating miRNAs are stable at room temperature for hours to days and hence particularly useful for triage in the field [18]. Over 80 miRNAs are readily detectable in serum/plasma and their changes after radiation can provide a readout of organ-specific responses [19,20]. Levels of specific circulating miRNAs in blood can change after radiation, as do the expression levels of messenger RNA (mRNA) [15,21]. Previous rodent studies have shown dose- and time-dependent alteration in serum levels of specific miRNA sequences after whole body irradiation (WBI) [15]. Particularly, a dose and time dependent decrease in miR-150 was noted, which was proposed as a novel approach for post-exposure dose estimation [22]. The lethal dose (LD_50_) for humans when untreated or with minimal supportive care is about 4 Gy [23, 24], which is significantly lower than LD_50_ for most rodents [25,26]. Therefore, validating the biomarker identity and dose response in non-human primates, with injury kinetics and immune response closer to humans [27] is highly desired. In this manuscript, we present the hematological parameters and circulating miRNA responses in non-human primates (NHP) exposed to total body irradiation at a low (1 Gy), sub-lethal (3 Gy) and a potentially lethal dose (6.5 Gy), evaluated at days 1, 3 and 7, the time points most relevant to triage in a radiological events [5,28,29]. The data shows a conserved pattern for changes in miRNAs in rodents and NHPs after whole body exposure. Like in rodents, circulating miR-150-5p was identified as the most sensitive biomarker for radiation biodosimetry. Additionally, several other evolutionarily and functionally conserved miRNAs were found to be altered in plasma collected from animals after whole body irradiation.

## Materials and Methods

### Whole body irradiation (WBI) study

Male and female rhesus macaques (Macaca mulatta) NHPs were exposed to 1 (N = 6), 3 (N = 8), or 6.5 Gy (N = 5). The day of irradiation was defined as Day 0. At the time of irradiation, the age of the animals ranged from approximately 2 to 5 years. On the day prior to irradiation (Day −1), the body weights ranged from 3.4 to 5.9 kg and from 3.8 to 6.5 kg, for males and females, respectively. All animals were declared healthy before inclusion and were evaluated for mortality and clinical signs (twice daily). Detailed examinations, including body weight and temperature, were performed, prior to animal assignment, during the week prior to irradiation, and on Days 1, 3, and 7. On day 0, the animals were subjected to a single uniform total body dose of gamma radiation from a ^60^Co source (Theratron 1000), with a targeted dose rate of 50 cGy· min^−1^. Sequential anteroposterior and posteroanterior exposures with whole body irradiation were used. Animals were not sedated during the irradiation and were breathing ambient air. Dosimeters (nanoDot^™^; Landuer Glenwood, IL) were placed on the animals for confirmation of exposure, however there was no real-time dosimetry performed via Farmer's chamber. In this study, all animals reached scheduled termination on Day 7. Blood samples were collected, without sedation or anesthesia, by venipuncture into a Vacutainer^®^ containing lithium heparin as an anti-coagulant for hematology three occasions prior to irradiation (within 2 weeks prior to irradiation) and daily for the first seven days after irradiation. Plasma was isolated and frozen (−80°C) prior to miRNA analysis.

### LPS study

In order to assess the specificity of miRNA biomarkers to ionizing radiation and to rule out or distinguish common systemic inflammation response indicators, 4 cohorts of 2 rhesus macaques (M. mulatta) NHPs each, with a body weight between 4.0–4.9 kg, were subjected to lipopolysaccharide (LPS) injections, known to induce a systemic cellular immune response [30]. Intravenous injection of LPS mimics inflammation and is used to assess marker specificity to irradiation. Briefly, on Day 1 of the study, an intravenous injection of LPS (Sigma Aldrich, Catalog number: 020m4062, serotype 0127:B8) reconstituted in sterile saline (0.9% sodium chloride) was administered (0.0, (vehicle-only sham), 0.7, 2.1, 2.8 mg/kg), without sedation or anesthesia. Blood samples were collected by venipuncture, without sedation or anesthesia, into a Vacutainer^®^ containing lithium heparin as an anti-coagulant for hematology once during the week prior to dosing and on Days 2 and 3. Blood samples for immunological assessment were processed to plasma. Plasma was prepared and frozen (−80°C) for miRNA analysis.

### Hematology

Hematology evaluations were performed on all animals, on three occasions prior to insult (irradiation or LPS, approximatively 2 weeks before) in order to assess the baseline, and daily thereafter. Among other parameters lymphocytes and neutrophils relative and absolute counts were measured (HemaVat analyzer).

### miRNA analysis by nanoString assay

An amplification-free, hybridization based direct digital counting method developed by NanoString Technologies was used for evaluation of changes in circulating miRNAs, as previously described [15]. However, in this assay, available human probes were used as miRNAs are evolutionarily conserved between human and NHPs. RNA was extracted from 200 μL plasma using Qiagen miRNeasy kit. After lysis, three synthetic oligonucleotides (spike-in oligos) were added. Purified RNA was eluted in 100 μL water and concentrated to 20 μL. The digital multiplexed nanoString nCounter miRNA expression assay was performed with RNA present in 3 μL (10–20 ng cell free RNA).

### Data analysis

Raw data from the nanoString assays were collected and normalized using internal positive spike-in controls to account for variability in the hybridization process. The data was further normalized by quantile normalization of the log_2_ counts to account for variability in the sample input. Data for miRNA sequences where the average log_2_ count did not exceed the measured limit of detection for the platform, determined by the average log_2_ counts of internal negative controls plus three times the standard deviation. To account for differences in abundance, counts for each miRNA sequence were scaled (zero mean, unit standard deviation). Using this data, miRNA sequences that apparently responded to radiation for each day (0, 1, 3, 7) were identified for each dose (1, 3, 7 Gy) by calculating the average absolute log_2_ fold change in counts and p-value (Student’s t-test) compared to the 0 Gy sham irradiated control. Sequences with an average absolute log_2_ fold change greater than 0.2 and a p-value < 0.05 were considered apparent responders. One-way analysis of variance (ANOVA) was performed to identify sequences that responded to radiation in a dose-dependent manner (p-value < 0.05) for each day. Day 0 animals were grouped based on the radiation that would be administered, but have received identical treatment at the time of blood draw.

### Animal study ethical statement

Procedures involving the care and use of animals in this study were reviewed and approved by the Institutional Animal Care and Use Committee (IACUC) of CiToxLAB North America prior to conduct of the studies (study numbers 2010–1113, 2011–0593, and 2011–0603). During the study, the care and use of animals was conducted in accordance with the principles outlined in the guidelines published by the Canadian Council on Animal Care and the Guide for the Care and Use of Laboratory Animals, an NRC publication. CiToxLAB North America’s facility is accredited by the Canadian Council on Animal Care and AAALAC. CiToxLAB North America’s OLAW animal welfare assurance number is A5525-01 and ChromoLogic and CiToxLAB had an approved inter-institutional agreement (#A7328-03).

Animals were group housed in accordance with CiToxLab standard operating procedures and recommendations of the Canadian Council on Animal Care committee in stainless steel monkey cages (0.39 m^2^ floor area per animal and 76.2 cm cage height).

Environmental enrichment and diet for the primates in this study was per CiToxLAB North America standard operating procedures which include television for at least one hour once a week, cages are equipped with a perch and various types of toys, and animals are provided treats including preapproved fruits and vegetables.

No animals were sacrificed for the LPS study.

At the end of the WBI studies, the animals were sedated with an intramuscular injection of a 0.10–-0.15 mL/kg combination of ketamine hydrochloride and acepromazine (100 mg/ml and 10 mg/ml, respectively) and then animals were euthanized by an intravenous overdose of sodium pentobarbital, followed by exsanguination. Doses of sedatives, anesthetic and other agents employed in terminal euthanasia were in accordance with CiToxLAB North America standard operating procedures. Animals found to be in un-relievable pain or distress per the following criteria were also euthanized per these procedures at the discretion of the Study Director and/or Clinical Veterinarian: respiratory distress (labored breathing, increased work-of-breathing); anorexia/Decreased appetite (complete anorexia for 3 days with deteriorating conditions based on the clinical examination, animal not taking nutritive supplement offered orally); significant weight loss defined as a decrement in excess of 20% of baseline weight for greater than 3 days despite nutritional support; decreased level of activity (recumbent (lateral recumbency) during an entire observation period, unresponsiveness to touch); acute, gross blood loss In excess of 20% estimated blood volume (source of acute blood loss not expected to respond to local measures, for example, gastrointestinal (emesis, per rectum)); generalized seizure activity; abnormal appearance (posture, rough coat, head down, exudates around eyes and nose, pallor, tucked abdomen and clinical appearance associated with abnormal vital signs); severe dehydration with hypothermia (decreasing rectal temperature reaching < 34.6°C and severely decreased activity level); or hyperthermia (rectal temperature >40.1°C and severely decreased activity level). Our analysis does not include data from animals that were prematurely euthanized for cause resulting from conditions described above as they were statistically insignificant (N = 1).

## Results

**Fig 1:**
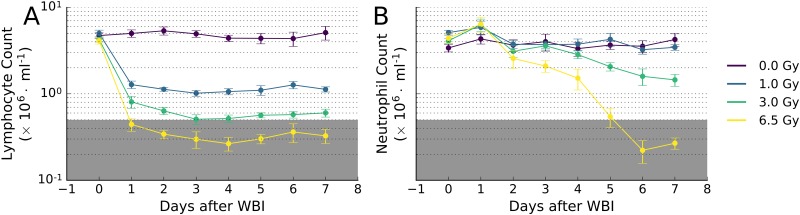
Time course of blood (A) lymphocyte and (B) neutrophil depression after graded doses of WBI in NHPs. The incidence of severe neutropenia at 7 days occurred only at doses exceeding 3 Gy. The lymphocytes responded with a rapid drop in counts within the first day after WBI. The neutrophils on the other hand showed a delayed response after 4 days, consistent with initial radiation sterilization of myeloid progenitors in the bone marrow and the turnover of mature cells. By day 7, a clear radiation dose-dependent depression of the blood cell counts was observed. The grey shaded regions in the plots correspond with severe lymphopenia and/or neutropenia (below 0.5 x 10^6^ cells/mL). These radiation dose-response relationships correspond to a predominant and significant feature of ARS.

**Fig 2:**
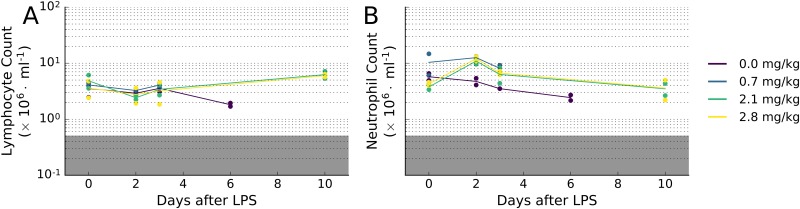
Time course of blood (A) lymphocyte and (B) neutrophil depression after doses of LPS in NHPs. Unlike WBI, LPS treatment did not induce significant changes in blood count. N = 2 for each condition.

In order to assess the individual tissue-specific response to radiation, we analyzed the time course of miRNA expression with blood lymphocyte and neutrophil depression (lymphopenia and neutropenia) after various dose of radiation and LPS. Careful analysis of NHP post-insult (WBI, LPS) lymphocyte and neutrophil levels provides an unbiased, quantitative criterion of hematopoietic response to the insult. In radiation related injury, dose related changes in total white cell counts are reflected in multiple changes in lymphocytes, and neutrophils through Day 7. Lymphocyte counts for animals in all irradiated groups decreased sharply and significantly (p-value < 0.0005) on day 1, relative to concurrent control group (0 Gy; Fig 1A), and same group pre-irradiation values (p-value < 0.0005). Lymphocyte counts generally continued to gradually decrease through day 3, then plateaued for the remainder of the study (day 7). Lymphocyte counts are highly sensitive to dose, and even the lowest dose of WBI (1 Gy) produced a significant decrease in the number of lymphocytes (p-value < 5 × 10^−7^). Neutrophil counts were less sensitive to WBI, with significant decreases apparent only after day 4 and only for doses ≥ 3 Gy (p-value < 0.005; Fig 1B). Severe lymphopenia and neutropenia (counts < 0.5 x 10^6^ cells/mL) were only observed in animals receiving 6.5 Gy of WBI (p-value < 0.05). LPS exposure did not significantly affect lymphocyte or neutrophil counts in NHPs, relative to animals receiving vehicle only (Fig 2).

To determine the effect of these stressors on circulating miRNA profiles, total RNA was extracted from frozen plasma samples after WBI or LPS treatment. The relative abundance of miRNAs was determined using an amplification-free, hybridization-based direct digital counting method, the nCounter miRNA expression assay (NanoString Technologies). Raw data from the nanoString assay consisting of the counts of 800 miRNA targets were collected and normalized using internal positive spike-in controls to account for variability in the hybridization process. The data was further normalized by quantile normalization of the log_2_ counts to account for variability in the sample input. Data for miRNA sequences that did not exceed the background, i.e. where the average log_2_ count did not exceed the measured limit of detection for the platform, determined by the average log_2_ counts of internal negative controls plus three times the standard deviation, was removed from subsequent analysis. To account for differences in abundance, each miRNA sequence was scaled (zero mean, unit standard deviation).

**Fig 3:**
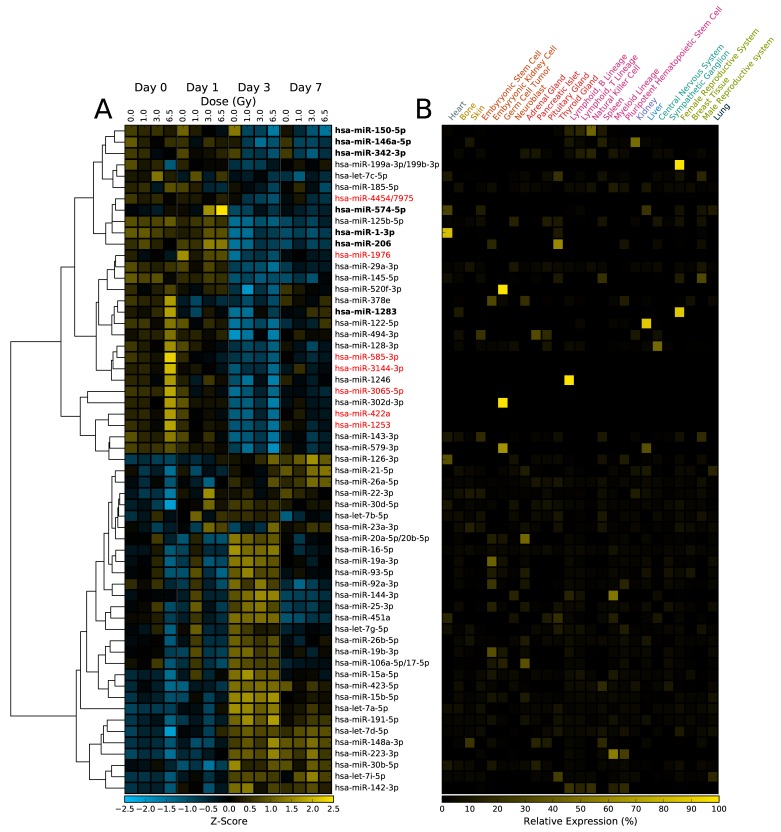
Heatmap of dose- and time-dependent changes in miRNA abundance in NHPs after exposure to ionizing radiation. (A) Of the approximately 800 miRNA sequences in the nanoString assay, less than 60 miRNAs were expressed above the limit of detection. Sequences that changed significantly (p-value < 0.05) are labeled in bold. (B) Expression profile of the miRNA for the indicated tissue types [31]. Expression data was not available for sequences labeled in red.

**Fig 4:**
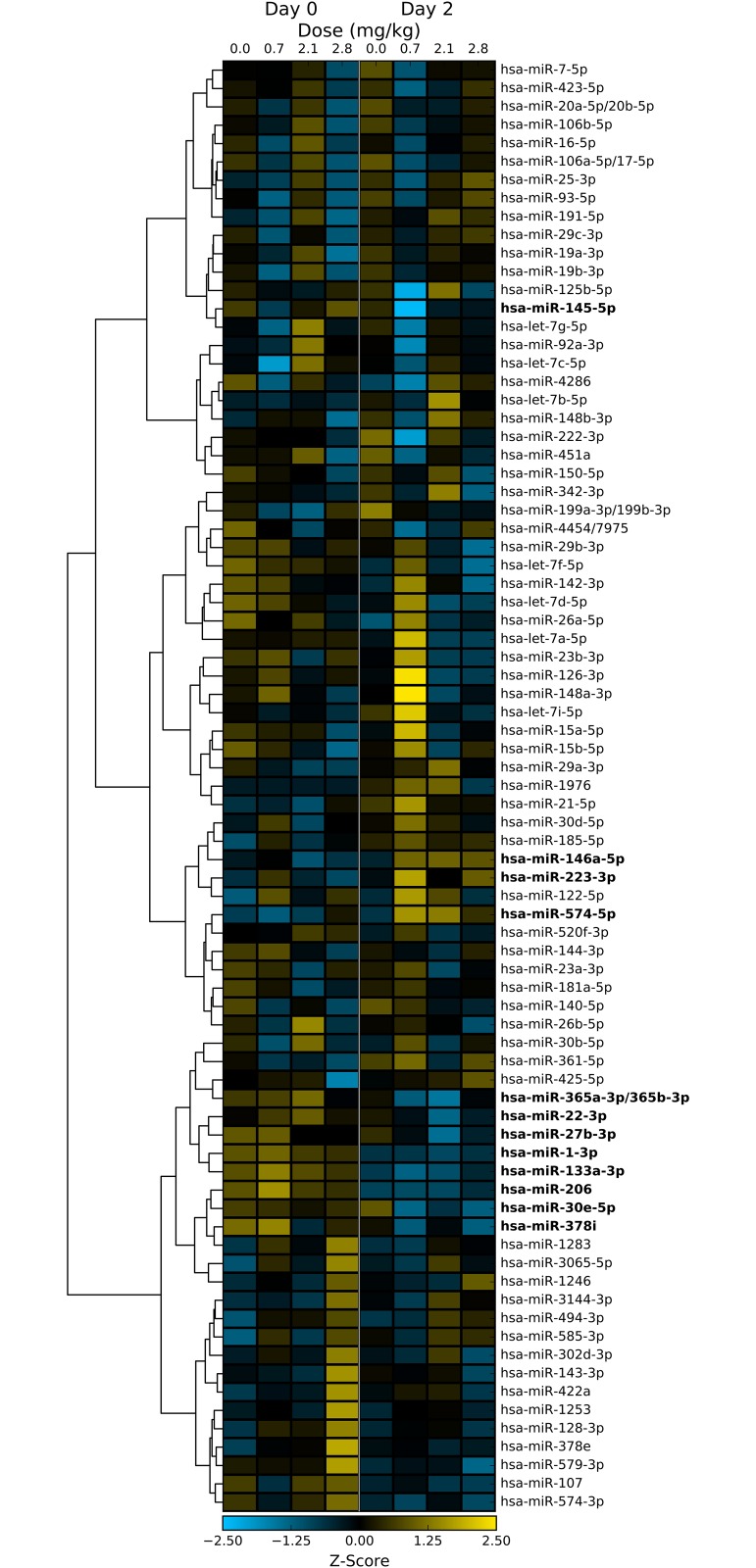
Heatmap of dose- and time-dependent changes in miRNA abundance in NHP after exposure to LPS treatment. Of the approximately 800 miRNA sequences in the nanoString assay, less than 60 sequences were expressed above the limit of detection. Sequences that changed significantly (p-value < 0.05) are labeled in bold.

**Fig 5:**
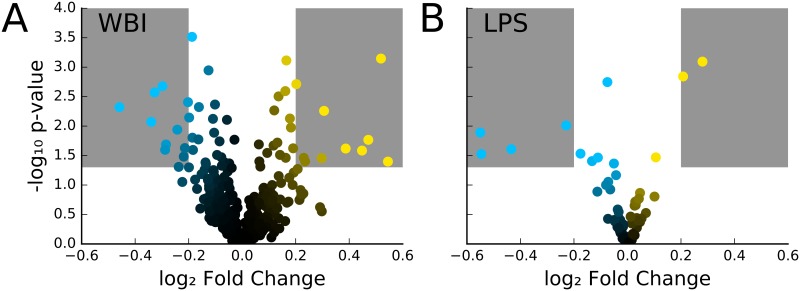
(A) Volcano plot for each day (1, 3, 7) comparing each radiation dose (1, 3, 6.5 Gy) to control (0 Gy). Regions in the plot shaded in gray indicate an average absolute log2 fold change > 0.2 and p-value < 0.05. As expected, because whole body irradiation doses in the study are at or below LD_50_, the overall miRNA expression profile in the body remains unchanged. From this plot, we pick the miRNAs that are significantly up or down regulated (shaded region) as candidate biomarkers for further analysis. (B). Volcano plot for comparing miRNA abundance two days after LPS insult for each dose (0.0, 0.7, 2.1, and 2.8 mg/kg, N = 2 for each condition) to pre-treatment (day 0). Regions in the plot shaded in gray indicate a |log2 fold change| > 0.2 and p-value < 0.05.

In the WBI samples, both dose-dependent and time-dependent trends can be observed for a number of miRNA sequences (Fig 3A). For example, the relative abundance of miR-574-5p significantly increases with dose on day 1 (p-value < 0.005), but does not show significant changes in abundance at day 3 or 7. On the other hand, miR-150-5p shows a dose-dependent decrease in abundance in all days, with strong decreases occurring in day 3 and 7 (Fig 3A). Many of these miRNA sequences display tissue-specific expression profiles, suggesting that miRNA profiles may be able to distinguish which tissues were affected by partial body exposure to ionizing radiation, a major unmet need in radiation exposure and triage planning (Fig 3B) [2,5]. WBI caused both increase and decrease in apparent abundance of circulating miRNA. Similarly, the LPS samples also show dose-dependent changes in circulating miRNA levels (Fig 4). LPS treatment caused significant increase in abundance of miR-146a-5p, miR-574-5p and decreased abundance of miR-1-3p, miR-133a-3p, miR-206, and miR-365a-3p/365b-3p. It is interesting to note that miR-574-5p expression levels were significantly changed in both LPS and WBI samples. As expected, several miRNA sequences showed both statistically significant (p-value < 0.05) and relatively large changes in abundance following WBI or LPS insult (Fig 5).

**Fig 6:**
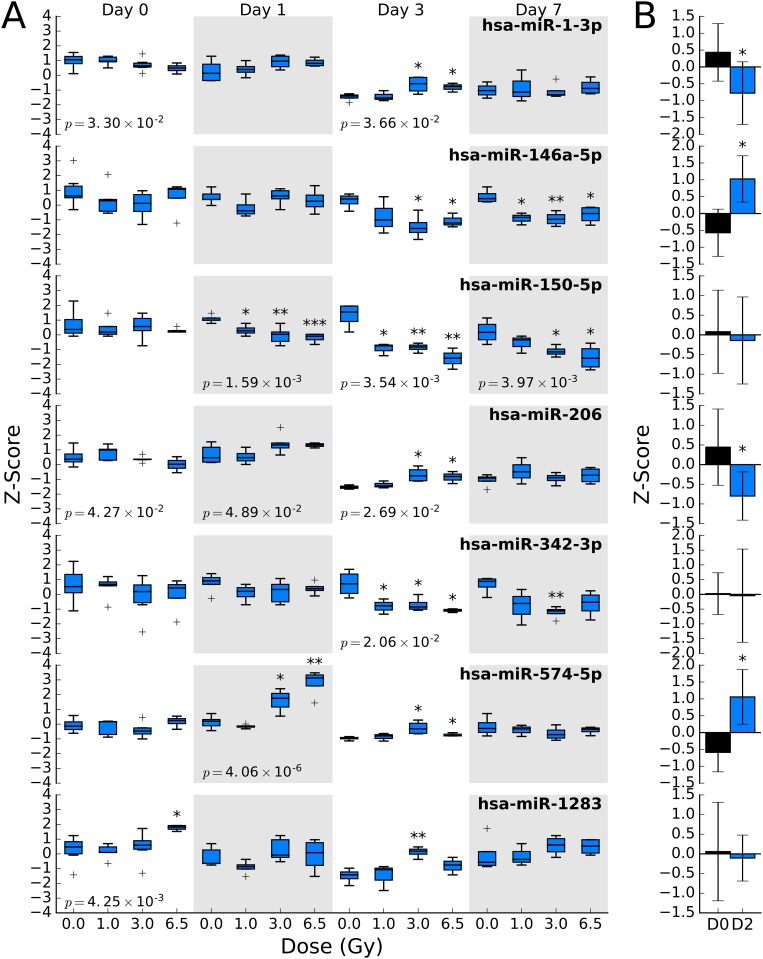
(A) Dose and time dependence of 7 miRNA sequences that significantly change (p-value < 0.05) after exposure to ionizing radiation at any dose or time point. One-way ANOVA was performed for each sequence at each day. The p-value for the analysis is shown on the box plot in cases where it is less than 0.05. Based on this analysis, hsa-miR-574-5p and hsa-miR-150-5p show significant dose response to WBI. (B) Corresponding changes in miRNA abundance after LPS treatment were significant for hsa-miR-1-3p, hsa-miR-146a-5p, hsa-miR-206, and hsa-miR-574-5p. * = p-value < 0.05, ** = p-value < 0.005, and *** = p-value < 0.0005.

These sequences, miR-1-5p, miR-146a-5p, miR-150-5p, miR-206, miR-342-3p, miR-574-5p, and miR-1283, had time- and dose-specific changes in abundance (Fig 6A). The data suggests that while the change in abundance of some miRNAs may be independent of dose (e.g. miR-1283), the study was able to identify specific, radiation sensitive, miRNA biomarkers viz. miR-150-5p. One-way ANOVA of the dose response of each sequence on each day suggests that miR-150-5p and miR-574-5p are sensitive to radiation (Fig 6A). Other ANOVA-positive sequences showed weaker dose dependence, similar to the observed variation for the sequence in the day 0 samples (e.g. miR-1-3p, miR-206, and miR-1283). The candidate biomarker sequences were compared to samples collected from the LPS study (Fig 6B). Few of the identified radiation biomarkers displayed no significant response to LPS treatment, indicating the response is specific to tissue damage resulting from radiation exposure as opposed to systemic inflammation. From the data, there exists an overlap between WBI and LPS miRNA expression profiles. This is consistent with the known systemic inflammatory response resulting from exposure to WBI [32].

**Fig 7:**
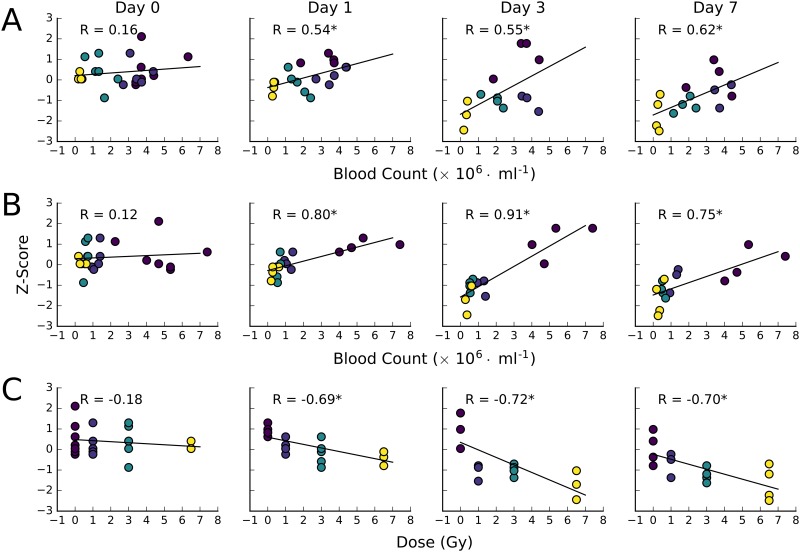
Correlation between neutrophil counts (A), lymphocyte counts (B), and administered dose (C) and miRNA abundance for miR-150-5p for each day. Points on the scatter plots are colored by the administered dose the animal received, as in C. Correlation coefficients (R) are displayed for each panel. An asterisk indicates the p-value of the fit is less than 0.05. The data shows that miR-150-5p expression profiles starting 1 day after WBI exposure is strongly correlated to the end-point (day 7) lymphocyte and neutrophil blood count, suggesting this biomarker could predict for the onset of ARS.

As neutrophil and lymphocyte counts on day 7 of the study (study endpoint) for each animal provides an unambiguous measure of the onset of ARS following WBI, the correlation between these values and miR-150-5p abundance was examined for each individual (Fig 7). We observed a strong correlation between neutrophil, lymphocyte, and radiation dose and miR-150-5p abundance. As expected, the correlation was strongest at day 3 and 7. The data shows that using the daily miRNA values, onset of neutropenia/lymphopenia can be predicted often starting from miRNA measurements made 1 day after irradiation.

## Discussion

As enacted in public law 108–276, Project Bioshield anticipates and considers exposure of the general population to a radiation/nuclear (RAD-NUC) terrorist event a major national threat. A RAD-NUC event could have many devastating forms, such as a nuclear detonation, use of a radiologic dispersive device (RDD, “dirty bomb”), attacks on a nuclear power plants/reactors, or the placement of radiation sources in public locations or in food. Furthermore, natural disasters like the 2011 earthquake and tsunami in Japan that resulted in the release of radiation into the environment further compound the risk to the general population. Radiation injury is directly proportional to the dose absorbed by specific tissue in the body as well as the individuals sensitivity to ionizing radiation [33]. As a result, the complexity of health problems ranges from ARS, that could lead to morbidity within days/weeks, to Delayed Effects of Acute Radiation Exposure (DEARE), that result in chronic conditions in lung, cardiovascular and kidneys over months. In order to manage and effectively treat a large population during a mass casualty RAD-NUC event when resources are severely strained,[1,28] a point-of-care (POC) tissue-specific biodosimeter that addresses specific risk to tissue associated with radiation is urgently needed. In the event of a mass casualty RAD-NUC event, it is anticipated that the resources available to triage the general population will be extremely limited. Exposure to WBI doses ≥ 2 Gy could lead to ARS. As a result, a POC device that measures the response of radiation-sensitive biomarkers is needed for early diagnosis and triage within 1–3 days after exposure. This would facilitate the treatment of mass casualty victims who are in need of care especially when emergency response services are severely strained. Circulating miRNA-based radiation biodosimetry holds significant promise as the assay is relatively straightforward and it requires only small volumes of blood obtainable without a phlebotomist, with the aid of commercially available lancets. It has been shown by us [15] and others [22] using mouse models, that miRNA-based assays are capable of taking into account inter-individual variations as the actual dose effect could vary depending on pre-existing conditions, inherent differences in immune responses and other confounders. Recent technological advances in exosome biology and liquid biopsy, coupled with our current studies in NHPs identifying specific biomarkers altered as function of WBI dose, will help in the development of an effective radiation biodosimeter that will be minimally invasive [31,34].

To identify radiation biomarkers, circulating miRNA levels were analyzed in NHPs after exposure to WBI. The efficacy of the dose regime, selected to reflect the doses expected to be encountered in a RAD-NUC event, was accessed by monitoring blood lymphocyte and neutrophil counts. Daily analysis of blood cell counts showed a predictable decrease in both lymphocytes and neutrophils following graded doses of WBI. A clear distinction in the time-course of lymphopenia and neutropenia was observed consistent with the different mechanisms of cell killing among these two cell populations. As mature lymphocytes are susceptible to the direct effects of radiation via interphase cell death (apoptosis), the decrease in cell counts are therefore rapid and seen within one day after WBI. On the other hand, mature neutrophils are predicted to be resistant to the direct effects of irradiation and are therefore not immediately affected. Nonetheless, they are continually required to be replaced by proliferating myeloid progenitors in the bone marrow, which are indeed sensitive to the sterilizing effects of ionizing radiation. Thus, neutrophil counts remain statistically indistinguishable from untreated animals during the first 4 days after WBI, but decrease rapidly thereafter. Depending on the radiation dose level, this can lead to the bone marrow syndrome associated with ARS when the neutropenia reaches a critical threshold (febrile neutropenia at 0.5 × 10^6^ cells/mL), necessitating interventive clinical management (e.g. blood transfusions, growth factor therapy and hematopoietic cell transplants). The radiation doses at which the animals passed the threshold for severe neutropenia (< 0.5 × 10^6^ cells/mL) were seen only at 6.5 Gy, consistent with the higher LD_20_ and LD_50_ estimates for rhesus macaques as compared to humans [27]. Severe hematological syndrome after radiation accidents is similarly reported to be at < 0.5 × 10^6^ cells/mL [35].

Thus, the WBI regimen used in this study is expected to elicit a range of responses up to the onset of ARS. Therefore, the resulting changes in the circulating miRNA profile are likely to be clinically relevant. Consistent with our previous report using rodent model of WBI, miR-150-5p exhibited the most robust dose and time dependent response. Parallel analysis of lymphocyte and neutrophil depletion kinetics with cell-free circulating miR-150-5p further validate the reliability of the later as an early biomarker for radiation biodosimetry. miR-150-5p is abundant in bone marrow and circulating lymphocytes, some of the cell-free circulating miR-150-5p are likely originated from bone marrow and the decrease in circulating miR-150-5p could be partly connected to the depletion in lymphocytes (Fig 5). As bone marrow is highly sensitive to radiation, the levels of circulating miR-150-5p will allow gauging the radiation dose in a range relevant to triage in radiological events. The levels of miR-150-5p in the circulating system could provide a direct readout of the level of functional marrow, and hence also provide a functional readout for hematological ARS. miR-150-5p is evolutionarily conserved, and previous studies have shown it as a key regulator of the transcription factor c-Myb, a regulator of hematopoiesis in multiple lineages [36,37]. An in vivo gain of function screen has identified miR-150-5p as an inhibitor of hematopoietic recovery upon marrow injury induced by the chemotherapeutic agent 5-flurouracil [38], where depletion of miR-150-5p resulted in enhanced hematopoietic recovery [38]. Also, studies using miR-150 knockout mice shows its effect on inhibition of inflammation and apoptotic response after myocardial infarction induced acute kidney injury [39]. Putative targets of miR-150 include Notch receptors and CXCR4, involved in multiple cellular processes including cellular growth, differentiation and cell mobilization and migration [40,41]. The interaction of miR-150 with CXCR4 level seems inversely correlated and it is likely that lowering of miR-150 would enhance the migration of lymphocytes in circulation. But, the overall decrease in bone marrow pool after irradiation itself would reduce the levels of circulating miR-150. Hence, the specific functional effect of its depletion on putative targets such as VEGF-A [42] is difficult to evaluate, and understanding those mechanisms would require additional research.

Another major outcome from the current study is the discovery of miR-574-5p as a novel early response biomarker, with clear dose response detectable at 24 h. A similar increase in circulating miR-574-5p abundance was observed in NHPs exposed to bacterial endotoxin LPS as well, suggesting that the plasma response here was associated with acute inflammatory response as a cause or effect. Circulating miR-574-5p could also be of viral origin, and the kinetics and response indicate possible nucleic acid-mediated activation of toll-like receptor signaling [43]. Moreover, multiple GU repeats present in the miR-574-5p sequence makes it a stronger agonist for TLR7/8 signaling [44]. Significant levels of miR-574-5p are expressed in lung, heart, and liver tissue [31], and radiation/LPS could also be stimulating the releases of extracellular vesicles from those organs, contributing to the observed increase in plasma levels on day 1. miR-146a is another molecule that exhibited significant plasma response after radiation as well as LPS treatment. Its increase after exposure to radiation as well as LPS treatment, suggest association with immune response [45–47]. Additional studies are needed to further understand the function of miRNAs that are altered, especially by specific tissues that are exposed to varying levels of ionizing radiation.

Studies on human cell lines have shown radiation dose response for several evolutionary conserved miRNAs, potentially modulating molecular processes such as angiogenesis and control of cell cycle [48–53]. However, the response of circulating cell-free miRNAs identified in the current study likely attributing to the differences in the release of extracellular vesicles from different radiosensitive organs as acute injury and/or inflammatory response [54]. The current study focused on the blood cell counts and circulating miRNAs in primates after exposure to low-LET gamma-rays. Additional studies are required to establish the robustness and kinetics of responses of these biomarker in primates after exposure to high-LET radiation, which would be likely component in the radiological events. Moreover, an earlier rodent study showed decrease in miR-150 in the whole blood after exposure to high-LET (^56^Fe) radiation [55].

Our study confirms the potential of circulating miRNAs as biomarkers for tissue-specific diagnostic screening that could be used to identify individuals potentially at risk of developing ARS. The dose range and time points selected in the current study are highly relevant to triage in radiological events. A multi-marker assay that include specific as well as systemic responders will allow post exposure dose estimation based on individuals’ own physiological response, thus reducing the effects due to several confounders. The sensitivity and kinetics of response varied with organs and detailed analysis and additional late time points following organ-targeted and organ protected irradiation are needed to develop a comprehensive panel for predicting the late response in organs such as lung, kidney and heart. Circulating miRNA biomarkers also could help monitor victims in the recovery stage and be an early assay to predict the onset and progression of delayed effects from exposure to radiation [56,57].

Biomarkers of normal tissue toxicity identified in this study will likely have impact in therapeutic radiation oncology as well. Whole body irradiation is commonly used for myeloablation in leukemia patients prior to bone marrow transplantation and there is significant concern of overdosing and/or underdosing because of underlying conditions and inherent variations in radiation sensitivity. Circulating biomarkers capable of providing early readout of systemic as well as organ specific responses will allow tailoring treatment based on individuals’ own physiological responses.

## Conclusions

To our knowledge this is the first study reporting changes in circulating miRNAs in NHPs at dose range relevant to triage in radiological events. While there are species specific differences in miRNA expression profiles, on the whole, there is a strong correlation in miRNA ARS biomarkers identified in this study and others reported in literature involving murine models. The relative ease in extracting and processing circulating miRNAs in a point of care and tissue specificity of miRNA expression together make circulating miRNA a promising candidate for radiation biodosimetry.

## Supporting Information

S1 File Lymphocyte and neutrophil counts of NHPs before and after WBI. In this file, the lymphocyte and neutrophil counts are expressed in millions per ml (× 10^6^ · ml^−1^). The dose of WBI is expressed in gray (Gy).
(CSV)

S2 File Lymphocyte and neutrophil counts of NHPs before and after LPS treatment. In this file, the lymphocyte and neutrophil counts are expressed in millions per ml (× 10^6^ · ml^−1^). The dose of LPS administered is expressed in mg/kg.
(CSV)

S3 File miRNA profiles for NHPs before and after WBI. Raw NanoString data for miRNA extracted from NHP plasma before and after WBI.
(CSV)

S4 File miRNA profiles for NHPs before and after LPS treatment. Raw NanoString data for miRNA extracted from NHP plasma before and after LPS treatment.
(CSV)

S5 File ANOVA statistics for miRNA sequences at different WBI doses. ANOVA statistics (F- and p-values) for each miRNA sequence for each day after irradiation (day 0, 1, 3, and 7).
(CSV)
